# Evaluating the personality structure of semi-captive Asian elephants living in their natural habitat

**DOI:** 10.1098/rsos.172026

**Published:** 2018-02-07

**Authors:** Martin W. Seltmann, Samuli Helle, Mark J. Adams, Khyne U Mar, Mirkka Lahdenperä

**Affiliations:** 1 Section of Ecology, Department of Biology, University of Turku, 20014 Turku, Finland; 2 Division of Psychiatry, University of Edinburgh, Royal Edinburgh Hospital, Edinburgh EH10 5HF, UK

**Keywords:** *Elephas maximus*, behavioural consistency, factor analysis, sex, questionnaires

## Abstract

Data on personality for long-lived, highly social wild mammals with high cognitive abilities are rare. We investigated the personality structure of Asian elephants (*Elephas maximus*) by using a large sample of semi-captive timber elephants in Myanmar. Data were collected during 2014–2017 using questionnaires, for which elephant riders (mahouts) rated 28 behavioural adjectives of elephants. Repeated questionnaires were obtained for each elephant from several raters whenever possible, resulting in 690 ratings of 150 female and 107 male elephants. We started by performing a confirmatory factor analysis to compare the fit of our data to a previously published captive elephant personality structure. Owing to a poor fit of this model to our data, we proceeded by performing explanatory factor analysis to determine the personality structure in our study population. This model suggested that personality in these elephants was manifested as three factors that we labelled as Attentiveness, Sociability and Aggressiveness. This structure did not differ between the sexes. These results provide the basis for future research on the link between personality and reproductive success in this endangered species and more generally, help to resolve the selective pressures on personalities in long-lived, highly social species.

## 1. Introduction

There is now well established evidence for personalities in non-human animals [1], with personality defined as consistent individual differences in behaviour across time and between contexts [2]. Animal personalities have numerous ecological and evolutionary implications, e.g. on life-histories, invasion and social evolution ([3] and references therein). In addition to consistent differences in state [4] and life-history trade-offs [5] leading to the evolution of personality, behavioural consistency can also coevolve with social responsiveness [6] and the specialization of individuals according to their social niche [7]. However, studies bridging human personality to that of other highly social, long-lived species are mostly restricted to a single taxon (primates) or occur only in captive settings and with limited sample sizes. The methods to describe personality in human and non-human studies also largely differ, hindering our ability to make generalizations concerning how the findings in humans may generalize to other long-lived social species beyond primates.

Two markedly different approaches exist for capturing between- and within-individual variation in personality: behavioural coding of the focal individual in natural or experimental settings (dominating research on animal personalities: 74% of studies [8], see also [9]), or trait ratings by experienced observers of the focal individual, both across differing environments and/or over time (including self-assessment in the case of research on human personality). Behavioural observations have the benefit of recording behaviours without subjective bias or the need of prior experience with the animal, and measurements are easily replicable within and between species [10]. However, the observational method also has some drawbacks [11] and cannot logistically be performed in all wild or environmental conditions. Ratings from experienced observers can serve as an integrated measurement of individual behaviour over a longer time period [10] and allow collection of a whole set of different behaviours instead of only the one or two items of interest [10]. Care has to be taken when designing questionnaires to avoid biases in ratings performed by humans and to prevent anthropomorphism. This is especially important when humans rate animals that focus on a different range of sensory and signalling modalities than their own (e.g. olfactory cues are more important in dogs than in humans), since this might lead to misinterpretations of behaviours. Using experienced raters to assess animal personality traits has proved to be a reliable and valid method (reviewed in [8]). Lately, there has been a call to broaden the scope of animal personality research by including more behavioural traits alongside commonly investigated traits (boldness, activity, exploration, sociability and aggression) [12], as these usually form sets of correlated traits called behavioural syndromes [13]. These multiple facets can be assessed by using the questionnaire approach, and this has been successfully applied to study personality differences in humans and other primates like chimpanzees, gorillas and orangutans [10]. Although the majority of animal personality research does not employ approaches more common in human personality research, there has lately been an increase in these kinds of studies [8,14–20].

Besides primates and cetaceans, elephants are an informative study system to link research on differences in personality in humans and non-human species. They present an independent evolutionary lineage of extensive longevity (up to 80 years), high sociality and cognitive abilities, and human-like life-history with long parental investment and reproduction over decades in family units, including relatively long post-reproductive lifespans [21–23]. Although there are differences between the social structures of some populations of African (*Loxodonta africana*) and Asian elephants (*Elephas maximus*), both genera live in complex fission–fusion family units [24]. Within and between these family units there are numerous interactive behaviours between individuals relating to predator avoidance, resource defence, information exchange and offspring care [21,22]. Family units usually consist of several related females and their offspring, whereas males disperse at adolescence [21,22]. Elephants have high social intelligence [25], the ability to solve cooperative tasks [26] and they show signs of self-recognition [27]. Consequently, interesting questions arise when considering their personality. The lifestyles of female and male elephants differ with females more social and gregarious than males, which in turn could lead to consistent sex-specific personality differences. These sex differences have previously been reported in a multitude of species [28–30], but have not been studied in detail in any non-primate long-lived social species. Characterizing personality dimensions in elephants and comparing those to results obtained from humans and other long-lived, social primates is thus of interest, as is linking those personality dimensions to wider results published across species on evolved similarities and differences between sexes.

In this study, we used trait ratings from questionnaires to unravel the personality structure of a large population of semi-captive Asian elephants working in the timber industry in Myanmar; an excellent system for looking at individual differences in a long-lived species. The elephants work during the day but are allowed to forage freely in the forest at night, thereby encountering wild conspecifics with which they interact. Offspring may be sired by wild bulls and there is no human assistance for reproduction or diet. Consequently, these timber elephants can express their natural behaviours to a large extent and are not confined to artificial environments, such as zoos or other forms of captivity, which are known to be costly for elephant welfare [31]. Previous studies on personality in Asian elephants were conducted only on individuals living in zoos (e.g. [32]), and their findings may not reflect personalities of individuals living in more natural settings. The only personality study with truly wild individuals focused on another species, the African elephant, where three or four experienced observers rated 26 behavioural adjectives which had previously been used and validated in other non-human species, though the sample size was low, with only 11 elephants rated [17]. We collected questionnaires from elephant riders (mahouts) rating 28 behavioural traits for a large number (*n* = 257) of semi-captive individuals they had experience with. Our first aim was to test whether the personality dimensions found for captive elephants [32] would apply to semi-captive Asian elephants. Since some differences between captive and semi-wild individuals were anticipated, and particularly if we found evidence for such differences in our data, we would proceed by performing exploratory factor analysis (EFA) on this set of 28 behavioural traits. As we were further interested in potential sex differences, we conducted confirmatory factor analysis (CFA) by sex based on the results of the EFA. Our study is the first to address the personality structure of Asian elephants living in their natural habitat.

## 2. Material and methods

### 2.1. Study population

Asian elephants are classified as endangered on the Red List of threatened species [33]. Myanmar has about 5000 semi-captive elephants [34], and over half of the semi-captive population is state-owned through Myanmar Timber Enterprise (MTE) and works in the timber industry. This population is ideal for the study of personality: its mortality rates [31], reproductive profiles [23] and social behaviours [35] resemble those of truly wild elephants and MTE maintain detailed log-books on every individual elephant. These log-books enable the recording of data for all registered elephants on registration number and name, birth origin (wild-captured or captive-born), date and place of birth (date estimated for wild-captured elephants), year and place of capture (if wild-captured); and sex. The age of captive-born elephants is known precisely while wild-caught elephants are aged by comparing their height and a range of physical features at capture with captive elephants of known age. Elephants work during state-set working hours under the guidance of a mahout in working groups of six elephants and a head mahout managing operations. At night individuals forage unsupervised in nearby forests where they mate both with each other and wild elephants, hence the ‘semi-captive’ definition of these animals. Around half of all timber elephants in the records were captured from the wild and tamed to work, but capture rates have reduced since 1997, and consequently the proportion of newly captured wild elephants working in timber camps has declined [34].

### 2.2. Collection of personality data

We assessed the personality of individual elephants from ratings by mahouts familiar with focal individuals. To ensure raters had sufficient exposure to the focal elephant, they were either the elephant's own mahout or the head mahout from the same working group of six. For the elephants' own mahouts, the median raters’ age was 24 years (interquartile range: 19–32 years; range: 14–60 years), and the median rater experience with their elephants was 2 years (interquartile range: 1–4 years; range: 1 day–22 years). The median age of rating head mahouts was 37 years (interquartile range: 32–46 years; range: 31–59 years), and their experience with their elephants was 4 years (interquartile range: 2–10 years; range: 2 months–33 years). Questionnaires were translated from English to the mahouts' native language (Burmese) by colleagues from Myanmar who have been working with elephants and are well familiar with the English language. Raters judged the frequency with which the focal elephant usually displays a particular behaviour or behavioural propensity on a 4-point scale, with 1 meaning ‘Very rarely’, 2 ‘Occasionally’, 3 ‘Quite a lot’ and 4 ‘Most of the time’. Questionnaires were based on a combined top-down and bottom-up approach, which has been shown to produce reliable results while facilitating cross-species comparisons [36]. Top-down adjectives were based upon an amalgamation of items and descriptions from previous elephant studies [32,37] and from primate questionnaires that have been previously correlated with behavioural observations [38]. Of our 30 original items, 15 items had been used in both elephant and primate personality studies, 11 items only in elephant personality studies and 3 items only in primate personality studies. Items used in previous elephant studies were, however, based on primate and dog personality studies, and most items from primate studies are ultimately based on human personality studies. We added the item ‘obedient’ to account for the working nature of these individuals, and two items (‘friendly’ and ‘social’) were separated into context-specific descriptors (behaviour towards the same versus opposite sex). To combine these top-down descriptors with a bottom-up approach, natural behaviours associated with the items were obtained from an ethogram and used wherever possible in the description to increase clarity and relevance of the ratings to wild behaviours, with each item given in the form of adjectives followed by a brief description. Since mahouts seemed to have difficulties in understanding the adjectives ‘sensitive’ and ‘gentle’, we removed those two items from further analysis, leaving us with 28 items (table 1). Mahouts were instructed not to discuss their ratings with other raters to ensure the independence of ratings. Questionnaires were conducted from March to June and in September 2014 and from March to April in 2015–2017.

**Table 1.** Items and their descriptions used to assess Asian elephant personality. Traits and descriptions were translated to Burmese before conducting questionnaires. Reliabilities of traits are shown by Fleiss' kappa values. Higher values represent a higher level of agreement between raters.

item	description	Fleiss’ kappa
active	The elephant moves around a lot, often at a fast pace and spends little time resting. Often vocalizes.	0.194
affectionate	Elephant seeks close relationship to others within their working group. This may include behaviours such as rubbing their forehead or body against another elephant or lying close to others while resting.	0.221
aggressive	Elephant causes harm or potential harm to other elephants; e.g. barks, charges, bites, kicks.	0.174
attentive	Elephant seems to listen (no ear flaps) closely to everything mahouts say or do.	0.143
confident	Elephant behaves in an assured manner. They make quick decisions and do not hesitate.	0.108
distractible	Elephant easily loses focus and has a short attention span.	0.136
dominant	Elephant behaves only as they please and becomes aggressive when interrupted; they may decisively intervene in social interactions; may push or displace others of a similar size or place trunk over another individual.	0.256
effective	Elephant successfully gets its own way for example by influencing the actions of other elephants.	0.267
fearful	Elephant reacts to threatening objects/situations in anxious or frightened manners; may grip the tip of their trunk to indicate nerves or vocalise to signify distress.	0.216
friendly	Elephant readily makes friends with people and elephants not part of their group. They are rarely hostile towards others of the same gender.	0.227
friendly (2)	Elephant readily makes friends with people and elephants not part of their group. They are rarely hostile towards others of the opposite gender.	0.152
impulsive	Suddenly gets excited and it is difficult to make the elephant calm.	0.222
inquisitive	Elephant explores new situations, objects, animals or people and tries to learn new things.	0.121
inventive	Elephant appears to act or play in novel, creative ways and may often create new tools for grooming etc.	0.145
mischievous	Elephant shows a fondness for causing trouble in a playful way such as spraying mucus by swinging his/her trunk.	0.223
moody	Elephant seems to be in a bad mood frequently, and displays mood swings often.	0.326
obedient	Elephant does as they are told by people with little to no resistance.	0.142
playful	Elephant initiates play and joins in when play is solicited.	0.090
popular	Elephant is sought out as a companion by others.	0.146
protective	Elephant shows concern for other elephants and often intervenes to prevent harm or annoyance from coming to them.	0.173
quitting	Elephant readily stops or gives up activities that have recently been started; requires encouragement to overcome barriers.	0.230
slow	Elephant moves in a relaxed, deliberate manner. The elephant is not easily hurried to complete tasks.	0.213
social	Elephant likes to make friends with other elephants of the same gender.	0.163
social (2)	Elephant likes to make friends with other elephants of the opposite gender.	0.177
solitary	Elephant prefers to spend time alone and does not seek out contact with other elephants.	0.173
subordinate	Elephant gives in readily to others of a similar size and acts as though lower in rank to other elephants, for example they will retreat or turn away in interactions.	0.195
timid	Elephant lacks self-confidence and easily becomes alarmed in unfamiliar social or non-social situations.	0.147
vigilant	Elephant carefully watches for possible dangers in the surroundings.	0.158

### 2.3. Study outline and sample size

This study included 257 elephants (150 females and 107 males) ranging in age from 3 to 76 years (females: 3–76, median: 29.5; males: 5–65, median: 16) from Pyinmana (Mandalay Division), Pinlaung (Shan State) and Kawlin and Katha (both in Sagaing Division). Of the 257 elephants, 63 elephants were wild-captured, 192 captive-born and 2 had unknown origin. The sample consisted of 316 mahouts rating 257 elephants, for a total of 690 ratings. Each mahout on average rated 1.7 times (range = 1–13) and each elephant was rated on average 2.7 times (range = 2–6).

### 2.4. Statistical data analysis

Statistical analyses were performed with the software R [39] and Mplus v. 8.0 [40]. To determine inter-rater reliability, we calculated Fleiss' kappa, suitable for ordinal categorical data (function kappam.fleiss from package irr [41] in software R). Values of −1 and 1 indicate perfect disagreement and agreement, respectively, while zero indicates agreement equal to chance. We summed ratings of the two mahouts per measurement occasion. We used sums instead of averages, because the summed ratings showed far less truncated distributions compared to averages. To test for sampling adequacy of the correlation matrix (function cor), we performed the Bartlett sphericity test (function cortest.bartlett), Steiger's test (function cortest.mat) and the Kaiser–Meyer–Olkin (KMO; function KMO) measure of sampling adequacy (all functions from package psych [42] in R software). The Bartlett sphericity test and Steiger's test were both significant (*p* < 0.01), showing that our correlation matrix was significantly different from a matrix in which traits are not correlated [43]. Furthermore, the overall KMO measure of sample adequacy was 0.97, exceeding the threshold value of 0.7 and hence fulfilling the criterion for the matrix being appropriate for further analyses (see [43] and references therein).

We started by examining the model proposed by Yasui *et al*. [32] for captive elephants using CFA and robust maximum-likelihood estimation [40]. Since our questionnaire and rated traits did not match exactly to the one used by [32], we could only use a subset of their original traits that corresponded to ours to test their model. The majority of our items conformed with the items used in [32] (18 out of 28) and the differences between the remaining 10 items were mostly based on differences related to the respective environment (zoo versus semi-captive) the elephants lived in. In this model, the factor ‘Dominance’ was measured by the items dominant, aggressive, moody and mischievous, the factor ‘Neuroticism’ by the items fearful, timid and vigilant, the factor ‘Agreeableness’ by the items friendly, social, gentle and affectionate, the factor ‘Impulsiveness’ by the items distractible, attentive, impulsive and quitting and the factor ‘Curious’ by the items inquisitive, playful, inventive and active. Pooled data and data separated by sex were analysed. Model fits to the data were examined using the Chi-square test (*χ*^2^) and the following fit-indices: the root mean square error of approximation (RMSEA), standardized root mean square residual (SRMR), and the comparative fit index (CFI) [44]. Both RMSEA and SRMR are badness-of-fit measures, where 0 indicates a perfect fit for the model. By contrast, in CFI a value approaching 1 indicates good model fit [44]. RMSEA has the added benefit of providing 90% confidence intervals for the estimate and it can be used to test the null hypothesis that the estimate is <0.05, indicating a good fit [44]. The rough cut-off values used to indicate a well-fitting model for SRMR and CFI are <0.08 and >0.95, respectively [44]. The major shortcoming of the *χ*^2^ test that should be non-significant for a well-fitting model is that it is an approximation and too powerful to detect trivial differences in cases of large sample size and high number of variables in the model, whereas all the fit-indices except the SRMR used here are insensitive to sample size [44].

In EFA with robust maximum-likelihood estimation, the determination of the number of factors to be extracted was evaluated using eigenvalues above one [45]. Items showing no salient loading (≥0.4) on any factor, strong cross-loadings (≥0.3) on more than one factor, or theoretical unsuitability on the present factor structure were deleted from the analysis step-by-step [45]. Oblique geomin rotation allowing correlated factors was used to calculate factor loadings. At every step, the interpretability of the present factor structure was evaluated and theoretically unrealistic factors and weak factors showing fewer than three salient loadings were deleted until the final factor structure was achieved. The fit of the final factor structure to the data was evaluated using the same fit criteria as in above CFA [45]. Finally, to examine potential sex differences, we examined the fit of the final model from EFA using CFA to the data by sex. As our data comprised repeated ratings of individual elephants within and between years, we used a design-based clustering method that corrects for non-independence of data points by adjusting parameter SEs without explicitly estimating this dependency [46]. Missing data were handled with full information maximum likelihood. The reliability of the extracted personality traits was estimated by calculating their composite reliability [47].

## 3. Results

### 3.1. Inter-rater reliability

We found evidence that mahouts' ratings captured elephant personality as there was slight overall agreement of trait ratings between raters (table 1). The values for Fleiss’ kappa ranged from 0.09 (playful) to 0.326 (moody) with a mean of 0.184. Of our 28 items, 10 showed fair agreement (greater than 0.20) and 18 showed slight agreement (greater than 0.01) [48]. Although the agreement between mahouts is arguably low, our inter-rater reliability measures are in line with previously published studies (e.g. [38,49]). However, low reliability of items should not inflict major problems, since factor analysis partitions variance in items into shared (caused by the factor) and unique (measurement error related to the specific item) variances. Moreover, summing the ratings of mahouts increases the reliability of this parcel above the reliability of a single measurement [50]. We, therefore, included all items in subsequent analyses.

### 3.2. The examination of the Yasui *et al*. model

Our results show that the model of a 5-factor personality structure in elephants was not supported by our data. By all the fit criteria used, the model where the sexes were pooled showed poor fit to the data (*χ*^2^ = 753.6, d.f. = 142, *p* < 0.0001; RMSEA (90% CIs) = 0.112 (0.104, 0.120), *p* < 0.0001; SRMR = 0.106; CFI = 0.824). The model also showed one inadmissible solution, as the correlation between the factors ‘Impulsiveness’ and ‘Neuroticism’ was larger than one (*r* = 1.022). The same conclusions were reached when considering only females (*χ*^2^ = 469.5, d.f. = 142, *p* < 0.0001; RMSEA (90% CIs) = 0.106 (0.095, 0.116), *p* < 0.0001; SRMR = 0.103; CFI = 0.849) or males (*χ*^2^ = 476.1, d.f. = 142, *p* < 0.0001; RMSEA (90% CIs) = 0.131 (0.118, 0.144), *p* < 0.0001; SRMR = 0.116; CFI = 0.771). Therefore, we decided to reject the 5-factor personality structure for our semi-captive Asian elephant population.

### 3.3. Exploratory factor analysis

We identified three distinct personality factors in Asian elephants. Table 2 presents the factor loadings of the retained 15 rated items on the three personality factors. We named the three personality factors Attentiveness, Sociability and Aggressiveness, according to the strongest loading item for Attentiveness (attentive) and Aggressiveness (aggressive). For Sociability, we decided to use the second strongest loading trait (social), since ‘Mischievousness’ (mischievous) might create confusion about the meaning of the factor. In general mischievous has a socio-negative meaning, but the factor does not reflect socio-negative behaviour (table 1 for description of mischievous). Attentiveness consisted of items that most likely reflect the responsiveness of the elephant towards its mahout and general movement (attentive, obedient, slow, vigilant, confident and active). Sociability related to positive social interactions (mischievous, social towards the same sex, playful, friendly towards people and elephants of the same sex, affectionate and popular). Aggressiveness was related to aggression and surliness (aggressive, dominant and moody). The three factors correlated with each other, with Attentiveness strongly correlating with Sociability and Aggressiveness showing an average correlation with Attentiveness and Sociability (table 3). Using fit criteria other than the *χ*^2^ test, the exploratory factor model for the sexes pooled showed acceptable fit to the data (*χ*^2^ = 125.8, d.f. = 63, *p* < 0.0001; RMSEA (90% CIs) = 0.054 (0.04, 0.067), *p* = 0.310; SRMR = 0.017; CFI = 0.979). The composite reliability values (indication item variance explained by the factor) for Attentiveness, Sociability and Aggressiveness were 0.95, 0.92 and 0.87, respectively.

**Table 2.** Structure of trait loadings on personality factors of elephants obtained from geomin rotation EFA (sexes combined). Salient loadings (≥0.4) are marked in bold.

item	attentiveness	95% CI	sociability	95% CI	aggressiveness	95% CI
attentive	**0**.**943**	(0.869,1.018)	−0.013	(−0.050,0.024)	−0.080	(−0.194,0.034)
obedient	**0**.**926**	(0.822,1.030)	−0.026	(−0.132,0.080)	−0.001	(−0.064,0.062)
slow	**0**.**810**	(0.667,0.953)	0.021	(−0.144,0.186)	0.004	(−0.084,0.092)
vigilant	**0**.**763**	(0.628,0.898)	0.103	(−0.058,0.264)	0.085	(−0.035,0.205)
confident	**0**.**728**	(0.569,0.887)	0.118	(−0.045,0.281)	0.022	(−0.060,0.104)
active	**0**.**700**	(0.533,0.867)	0.208	(0.038,0.379)	−0.011	(−0.066,0.044)
mischievous	−0.023	(−0.227,0.181)	**0**.**824**	(0.626,1.022)	0.017	(−0.116,0.150)
social	−0.029	(−0.205,0.147)	**0**.**807**	(0.580,1.034)	0.094	(−0.133,0.321)
playful	0.095	(−0.119,0.309)	**0**.**771**	(0.550,0.993)	−0.034	(−0.179,0.111)
friendly	0.085	(−0.142,0.312)	**0**.**758**	(0.523,0.993)	−0.073	(−0.251,0.105)
affectionate	0.098	(−0.104,0.299)	**0**.**712**	(0.500,0.924)	0.050	(−0.160,0.260)
popular	0.003	(−0.034,0.040)	**0**.**666**	(0.529,0.803)	0.202	(0.008,0.396)
aggressive	−0.012	(−0.038,0.014)	0.111	(−0.146,0.368)	**0**.**829**	(0.688,0.970)
dominant	0.051	(−0.153,0.255)	0.042	(−0.209,0.293)	**0**.**777**	(0.624,0.930)
moody	0.133	(−0.069,0.335)	−0.036	(−0.134,0.062)	**0**.**727**	(0.566,0.888)

**Table 3.** Correlation coefficients between the three extracted factors (sexes combined).

	attentiveness	sociability	aggressiveness
attentiveness	1.00		
sociability	0.756		
		1.00	
aggressiveness	0.536	0.425	1.00

### 3.4. Confirmatory factor analysis by sex

To confirm the fit of our 3-factor model obtained from EFA (§3.3) for each sex separately, we conducted CFA for males and females separately. This model showed good fit to the data for both females (*χ*^2^ = 141.6, d.f. = 87, *p* < 0.001; RMSEA (90% CIs) = 0.055 (0.038, 0.071), *p* = 0.293; SRMR = 0.036; CFI = 0.970) and males (*χ*^2^ = 109.7, d.f. = 87, *p* = 0.05; RMSEA (90% CIs) = 0.043 (0.000, 0.067), *p* = 0.651; SRMR = 0.036; CFI = 0.982). Thus personality manifests as three factors, both in female and male elephants. The structure of trait loadings per factor differed slightly between the sexes. Table 4 presents the structure of trait loadings on personality factors of female elephants and table 5 presents the structure of trait loadings on personality factors of male elephants, both obtained by CFA. Tables 6 and 7 present the correlations between the three factors in females and males, respectively. For females, the composite reliability values for Attentiveness, Sociability and Aggressiveness were 0.95, 0.92 and 0.88, respectively and for males 0.95, 0.92 and 0.84, respectively.

**Table 4.** Structure of trait loadings on personality factors of female elephants obtained from CFA. Highest loading values are marked in bold.

item	attentiveness	95% CI	sociability	95% CI	aggressiveness	95% CI
attentive	1.619	(1.462,1.776)				
obedient	1.709	(1.554,1.864)				
slow	1.625	(1.435,1.815)				
vigilant	**1.775**	**(1.622,1.928)**				
confident	1.549	(1.390,1.708)				
active	1.595	(1.430,1.760)				
mischievous			1.303	(1.121,1.485)		
social			1.409	(1.213,1.605)		
playful			1.405	(1.219,1.591)		
friendly			1.336	(1.142,1.530)		
affectionate			**1.484**	**(1.335,1.633)**		
popular			1.189	(1.024,1.354)		
aggressive					0.976	(0.823,1.129)
dominant					1.032	(0.836,1.228)
moody					**1.126**	**(0.916,1.336)**

**Table 5.** Structure of trait loadings on personality factors of male elephants obtained from CFA. Highest loading values are marked in bold.

item	attentiveness	95% CI	sociability	95% CI	aggressiveness	95% CI
attentive	**1.843**	**(1.649,2.037)**				
obedient	1.751	(1.559,1.943)				
slow	1.631	(1.411,1.851)				
vigilant	1.833	(1.631,2.035)				
confident	1.653	(1.439,1.867)				
active	1.802	(1.582,2.022)				
mischievous			1.21	(1.022,1.398)		
social			1.44	(1.228,1.652)		
playful			**1.463**	**(1.243,1.683)**		
friendly			1.431	(1.206,1.656)		
affectionate			1.419	(1.221,1.617)		
popular			1.21	(0.990,1.430)		
aggressive					**1.086**	**(0.902,1.270)**
dominant					1.024	(0.816,1.232)
moody					0.984	(0.768,1.200)

**Table 6.** Correlation coefficients between the three extracted factors (females).

	attentiveness	sociability	aggressiveness
attentiveness	1.000		
sociability	0.878		
		1.000	
aggressiveness	0.630	0.588	1.000

**Table 7.** Correlation coefficients between the three extracted factors (males).

	attentiveness	sociability	aggressiveness
attentiveness	1.000		
sociability	0.737		
		1.000	
aggressiveness	0.580	0.540	1.000

## 4. Discussion

Personality investigations in large, long-lived, highly social mammals other than primates are rare, while methodological differences between human and non-human studies also limit generalizations. We used a unique and large dataset on semi-captive Asian elephants in Myanmar to provide evidence that elephants also have a complex personality structure, though it is not associated with the sex of the individual. We identified three personality factors in Asian elephants: Attentiveness, Sociability and Aggressiveness.

Irrespective of sex, our data showed poor fit for the elephant personality structure presented in Yasui *et al*. [32] using zoo elephants. This could be for several reasons. First, we could not examine the exact factor structure proposed by Yasui *et al*. because we did not have exactly the same traits available as they had in their study. However, reflective items in factor models, where a factor is assumed to cause variation in items, are thought to be exchangeable, meaning that dropping items should not change the meaning of a factor. Second, their study was conducted on captive individuals living in zoos, whereas our study involved elephants living in their natural habitat surrounded by a large number of semi-captive and wild elephants, and being able to express more natural behaviours than elephants confined to artificial zoo environments. Third, their study comprised pooled data from two similar but nonetheless different species, African and Asian elephants. However, their Dominance factor is very similar to our Aggressiveness factor. Dominance includes, among others, the traits dominant, aggressive and moody, which represent Aggressiveness in our study. This may indicate that this factor is an essential part of elephant personality that can be found independently of differences in the environment or differences between closely related species. Dominance also included the trait mischievous, which in our model loaded on Sociability, a factor describing positive social interactions rather than aggressiveness or dominance. However, mischievous did not load very highly on Dominance (0.48) [32], whereas here mischievous was the strongest loading trait on the Sociability factor (0.82), which indicates that this item is more likely to be seen as a socio-positive behaviour rather than having an agonistic character. All other traits loading on Sociability and Attentiveness in our model are distributed over several factors in the Yasui *et al*. model, making those factors incomparable. We therefore suggest that the personality structure found by Yasui *et al*. may not be applicable for elephant populations living in more natural environments, such as our semi-captive population in Myanmar.

The first personality dimension detected in our study was Attentiveness. This factor seems mostly related to the response of the elephant towards commands from mahouts and how it acts in and perceives its environment in general. Several traits in this factor concern elephant–mahout interactions (tables 1 and 2). This is the first time a personality factor like Attentiveness has been observed in elephants. The traits loading onto Attentiveness have been previously associated with separate factors like Impulsiveness, Neuroticism and Curiosity in zoo elephants [32] and Leadership and Playful in wild African elephants [17]. Attentiveness might be a unique factor in our study population and describes the working nature of the timber elephant population; comparisons with other working populations such as domestic horses or search dogs might offer interesting future research avenues.

The second factor we called Sociability. Elephants scoring high on Sociability seek closeness to other elephants and show positive social behaviour towards them, outsiders and humans, and they both seek out and are sought after by others as social partners. Elephants live naturally in complex fission–fusion family units which are part of a multi-tiered elephant society (e.g. [21,22]) and experience complex social interactions over their lifetime. The existence of a social personality factor in such a group-living social species thus would be expected. Interestingly, a factor describing sociability has not only been found in elephants [17,32,37], but also in other highly social primates including humans [10,30]. To corroborate the generality of a sociability factor in social species, it would be of high value to investigate non-social species for the presence or absence of such a factor, as well as more studies on other highly social independently evolved mammal lineages. Our study on Asian elephants thus provides an important point of comparison in a previously primate-centric field.

Evidence for our third personality factor, Aggressiveness, is ambiguous in elephants. The traits loading on the factor Aggressiveness in our model—behaving aggressively towards conspecifics, interfering in social interactions and displacing other individuals, and displaying mood swings—are largely in concordance with those identified in zoo elephant populations [32]. As mentioned earlier, our Aggressiveness factor resembled the Dominance factor found in [32]. By contrast, a study on 11 wild African elephants could not identify an aggressiveness factor [17]. Their trait aggressive loaded negatively on a Gentle factor and did not form a separate factor with other traits describing agonistic or dominant behaviours. The authors explain the lack of dominance with the strong positive social behaviour and the rarely occurring aggression between family members. However, dominance as a trait itself was not included in the principal component analysis in their study. On the other hand, dominance is the outcome of two individuals interacting and not a personality trait *per se*, and dominance can be related to personality in a context-dependent way in the wild (e.g. [51,52]). The trait dominant which we used in our questionnaire is perhaps not applicable in elephants or should at least be renamed. Nevertheless, the existence of a factor that comprises agonistic behaviour in Asian elephants is not surprising; e.g. when family units meet at a water hole, social interactions are mainly characterized by dominance bouts [21]. Social interactions related to e.g. dominance hierarchies are also important in many non-human primate societies [53], and in those species a similar factor in personality has also been found [10].

Our results suggest there is no factor describing ‘Neuroticism’ or fearfulness [32,37] in our elephant population. Neuroticism or Fearful are factors generally associated with behaviours describing fear and insecurity. In elephants and primates, such a factor is often found in zoo populations [32,37,54]. Perhaps living in a semi-captive natural environment might explain why we fail to discover a ‘Neuroticism’ factor in our population, as our elephants do not suffer from the full confinements of captivity. Similarly, a study on wild African elephants did not find evidence for the existence of a ‘Neuroticism’ factor, and the authors argue that it might be due to the elephants living in the wild undisturbed by human observers [17]. On the other hand, humans who do not live in confined spaces possess a ‘Neuroticism’ factor [55], and other non-human animals commonly express fearful behaviour in the wild (e.g. [56]), not just in zoo environments. Another explanation for the lack of a ‘Neuroticism’ factor might be that younger mahouts might not have been able to assess items related to this factor as reliably as older mahouts. For chimpanzees of Gombe National Park, the reliability for Neuroticism was higher for older field assistants than for younger field assistants [57]. The lack of a fearfulness factor in our population still remains a puzzle that needs to be addressed in future studies.

We decided to name factor 1 (Attentiveness) and 3 (Aggressiveness) after the item loading strongest on the factor (attentive and aggressive, respectively; table 2). Factor 2 (Sociability) is better represented by the item with the second strongest loading (social) than by the item with the strongest loading (mischievous; table 2). For comparative studies, consistent terminology for assessing traits is important to make meaningful comparisons, which is one reason why we decided to use items previously used in elephant studies [17,32,37]. However, even though we found similarities between previous studies on elephants and primates, we decided to take a conservative approach when naming the factors. We did not call our factors, e.g. Agreeableness, Dominance or Neuroticism [32,55] as we do not want to force similarities between studies with the use of the same, possibly misleading labels: our factors did not correspond completely with previous findings (as our CFA results suggest for the Yasui *et al*. model [32]) and we believe that our factor names are more appropriate for our results. Of course, the behavioural traits we used do not capture the complete picture of complex elephant behaviours. For instance, none of our items have assessed behaviours regarding cooperation [26], maternal care, mate choice or foraging [21,22]. In our population, these behaviours occur to a large extent unobserved during the elephants' free time and at night. Therefore, we cannot say with certainty that these three personality factors identified here depict a complete account of Asian elephant behavioural variation, and it might well be that these three factors are more complex, or that there are additional factors to find. Attentiveness seems to be a relevant factor to describe individual differences in elephant–mahout interactions in our working population: some elephants seem to be better at responding to mahouts’ commands and at fulfilling their work duties, whereas other individuals are often regarded as less able for certain tasks. Also, since elephants spend a lot of time together in work groups, distinct friendship groups (unpublished data) and family groups [35] and frequently interact with human handlers, variation in Sociability and Aggressiveness seems plausible.

A three-factor personality structure in Asian elephants may be compared to similar personality structures identified in elephants [17,37] and non-human primates [38,58]. The similarities in personality factors for humans and non-human primates might stem from common ancestral personality dimensions [49]. By contrast, any similarities between elephants and other highly social, long-lived mammals are more likely to be the result of convergent evolution and might be linked to shared patterns of sociality and similar selective pressures acting on the evolution of personality structure [6,7]. An alternative explanation is that the similarities are a methodological artefact: studies investigating the personalities of humans, other primates and elephants have all employed similar questionnaires [59]. However, the structure of the three personality factors we found in Asian elephants was not identical to what has previously been found in elephants and non-human primates, possibly as we used experienced raters who sometimes had a decades-long working history with their elephants, and for many elephants ratings were obtained from multiple raters. We, therefore, propose that living in a highly social environment, being long-lived and having high cognitive abilities constitute similar conditions in elephants and other highly social, long-lived mammals for selection to shape behaviour and personality in similar but not necessarily analogous ways. Against our expectation, we found no statistical evidence for sex differences in personality. This is somewhat surprising, since the social lives of female and male elephants differ in their natural environment. Female Asian elephants live in small family units with strong bonds between the group members and group cohesion is of high importance [21], while little is known about the social life of male Asian elephants: they often live solitary or in loose male groups and are more likely to be associated with females in oestrus during musth [21]. Close relationships with other individuals may therefore be less important for males than for females. Regardless of the differences between their social lives, female and male Asian elephants in our semi-captive population do not show overall personality differences. Not all highly social species express strong or similar patterns in sex-specific differences in personality [60]. Living and working together in timber logging camps may weaken any potential sex effects in observable personality differences in our population; the elephants are allowed to roam the forest freely at night, but are returned to the camps for the day during which females and males spend most of their time together [34]. It is noteworthy, however, that despite the lack of general sex differences in personality structure, elephants showed subtle sex-specific differences in the structure of loadings within each factors.

Mahouts usually work together with their focal elephant for many years, often for their whole life. Therefore, mahouts gain profound knowledge about their elephant's behaviour, and probably nobody else could assess these elephants better than the mahouts. However, future work should investigate the effect of raters' personality, personal attitude and experience on the ratings of a subject's personality. There is evidence that variation in pet owners' personality is related to pets’ behaviour [61,62]. Therefore, there is reason to believe that the mahouts' own personalities might relate to how they rate their elephant's personality or even affect their elephant's behaviour or personality itself. For example, mahouts with lower Neuroticism and higher Conscientiousness [55] scores might be more able to control their elephants, which might in turn lead to higher ratings in traits related to elephant Attentiveness. Furthermore, mahouts with higher scores on Openness [55] might be more willing to work with new elephants or adapt to new training methods. Finally, mahouts with higher score in Agreeableness [55] might behave more compassionately towards their elephant. It is also conceivable that mahouts that have had bad experiences with certain elephants might rate them with an unconscious bias towards them being e.g. more aggressive or less social than the elephants actually are. Future investigations should assess mahouts' personality and this information might shed light on the potential effects of personality in human–elephant interactions.

Our results have several important implications. First, as our questionnaire approach captured the multifaceted nature of elephant personality, our results add support for incorporating questionnaires in behavioural ecology research as previously advocated [1]. Our findings will enable further investigations of the relationship between personality, life-history and fitness in elephants and will help to facilitate management of the Myanmar timber elephant populations. Using a large sample of semi-captive Asian elephants which show mortality rates [31], reproductive profiles [23], social behaviours [35] and life conditions similar to wild elephants, we provide evidence for a sex-independent three-factor personality structure. Understanding elephant personality also supports further research and conservation efforts for the benefit of Asian elephants and other highly social long-lived mammals. Knowledge about the elephants’ personalities could benefit population management efforts, e.g. through increased reproductive success and in efforts to make the population self-sustaining [1], whereas elephant keepers might benefit in terms of better work efficiency as successful cooperation may be linked to certain complementary combinations of personality types [63]. Furthermore, individuals of certain personality types can differ in trainability [64]. Personality has also been linked to subjective well-being in animals [10] and this personality assessment in Asian elephants provides a vital data point for comparative personality research and forms the foundation for more studies into selective pressures generating and maintaining personality differences in wild animal populations.

## Supplementary Material

Elephant personality data

## Data accessibility

The data are provided as electronic supplementary material ‘electronic supplementary material_1_SeltmannEtAl_data’.

## References

[1] Carere C, Maesripieri D (eds). 2013 Animal personalities: behavior, physiology, and evolution. Chicago, IL: The University of Chicago Press.

[2] Réale D, Reader SM, Sol D, McDougall PT, Dingemanse NJ 2007 Integrating animal temperament within ecology and evolution. Biol. Rev. 82, 291–318. (doi:10.1111/j.1469-185X.2007.00010.x) 17437562 10.1111/j.1469-185X.2007.00010.x

[3] Wolf M, Weissing FJ 2012 Animal personalities: consequences for ecology and evolution. Trends Ecol. Evol. 27, 452–461. (doi:10.1016/j.tree.2012.05.001) 22727728 10.1016/j.tree.2012.05.001

[4] Sih A, Mathot KJ, Moirón M, Montiglio PO, Wolf M, Dingemanse NJ 2015 Animal personality and state-behaviour feedbacks: a review and guide for empiricists. Trends Ecol. Evol. 30, 50–60. (doi:10.1016/j.tree.2014.11.004) 25498413 10.1016/j.tree.2014.11.004

[5] Wolf M, van Doorn GS, Leimar O, Weissing FJ 2007 Life-history trade-offs favour the evolution of animal personalities. Nature 447, 581–584. (doi:10.1038/nature05835) 17538618 10.1038/nature05835

[6] Wolf M, Van Doorn GS, Weissing FJ 2011 On the coevolution of social responsiveness and behavioural consistency. Proc. R. Soc. B 278, 440–448. (doi:10.1098/rspb.2010.1051) 10.1098/rspb.2010.1051 PMC3013403 20739321

[7] Bergmüller R, Taborsky M 2010 Animal personality due to social niche specialisation. Trends Ecol. Evol. 25, 504–511. (doi:10.1016/j.tree.2010.06.012) 20638151 10.1016/j.tree.2010.06.012

[8] Gosling SD 2001 From mice to men: what can we learn about personality from animal research? Psychol. Bull. 127, 45–86. (doi:10.1037/0033-2909.127.1.45) 11271756 10.1037/0033-2909.127.1.45

[9] Freeman H, Gosling SD, Schapiro SJ 2011 Comparison of methods for assessing personality in nonhuman primates. In Personality and temperament in nonhuman primates. Developments in primatology: progress and prospects (eds Weiss ML, King AJ, Murray L), p. 346 New York, NY: Springer.

[10] Freeman HD, Gosling SD 2010 Personality in nonhuman primates: a review and evaluation of past research. Am. J. Primatol. 72, 653–671. (doi:10.1002/ajp.20833) 20568079 10.1002/ajp.20833

[11] Carter AJ, Feeney WE, Marshall HH, Cowlishaw G, Heinsohn R 2013 Animal personality: what are behavioural ecologists measuring? Biol. Rev. 88, 465–475. (doi:10.1111/brv.12007) 23253069 10.1111/brv.12007

[12] Koski SE 2014 Broader horizons for animal personality research. Front. Ecol. Evol. 2, 70 (doi:10.3389/fevo.2014.00070)

[13] Sih A, Bell AM, Johnson JC, Ziemba RE 2004 Behavioural syndromes: an integrative overview. Q. Rev. Biol. 79, 241–277. (doi:10.1086/422893) 15529965 10.1086/422893

[14] Kuczaj SA II, Highfill L, Byerly H 2012 The importance of considering context in the assessment of personality characteristics: evidence from ratings of dolphin personality. Int. J. Comp. Psychol. 25, 309–329.

[15] Jones AC, Gosling SD 2005 Temperament and personality in dogs (*Canis familiaris*): a review and evaluation of past research. Appl. Anim. Behav. Sci. 95, 1–53. (doi:10.1016/j.applanim.2005.04.008)

[16] Lloyd AS, Martin JE, Bornett-Gauci HLI, Wilkinson RG 2007 Evaluation of a novel method of horse personality assessment: rater-agreement and links to behaviour. Appl. Anim. Behav. Sci. 105, 205–222. (doi:10.1016/j.applanim.2006.05.017)

[17] Lee PC, Moss CJ 2012 Wild female African elephants (*Loxodonta africana*) exhibit personality traits of leadership and social integration. J. Comp. Psychol. 126, 224–232. (doi:10.1037/a0026566) 22905995 10.1037/a0026566

[18] Gartner MC, Powell D 2012 Personality assessment in snow leopards (*Uncia uncia*). Zoo Biol. 31, 151–165. (doi:10.1002/zoo.20385) 21455952 10.1002/zoo.20385

[19] Gosling SD 1998 Personality dimensions in spotted hyenas (*Crocuta crocuta*). J. Comp. Psychol. 112, 107–118. (doi:10.1037/0735-7036.112.2.107) 9642781 10.1037/0735-7036.112.2.107

[20] Gartner MC 2015 Pet personality: a review. Pers. Individ. Dif. 75, 102–113. (doi:10.1016/j.paid.2014.10.042)

[21] Sukumar R 2003 The living elephants: evolutionary ecology, behavior, and conservation. Oxford, UK: Oxford University Press.

[22] Moss CJ, Croze H, Lee PC (eds). 2011 The Amboseli elephants: a long-term perspective in a long-lived mammal. Chicago, IL: The University of Chicago Press.

[23] Lahdenperä M, Mar KU, Lummaa V 2014 Reproductive cessation and post-reproductive lifespan in Asian elephants and pre-industrial humans. Front. Zool. 11, 54 (doi:10.1186/s12983-014-0054-0) 25183990 10.1186/s12983-014-0054-0 PMC4144032

[24] de Silva S, Wittemyer G 2012 A comparison of social organization in Asian elephants and African savannah elephants. Int. J. Primatol. 33, 1125–1141. (doi:10.1007/s10764-011-9564-1)

[25] Bates LA, Sayialel KN, Njiraini NW, Poole JH, Moss CJ, Byrne RW 2008 African elephants have expectations about the locations of out-of-sight family members. Biol. Lett. 4, 34–36. (doi:10.1098/rsbl.2007.0529) 18055407 10.1098/rsbl.2007.0529 PMC2412944

[26] Plotnik JM, Lair R, Suphachoksahakun W, de Waal FBM 2011 Elephants know when they need a helping trunk in a cooperative task. Proc. Natl Acad. Sci. USA 108, 5116–5121. (doi:10.1073/pnas.1101765108) 21383191 10.1073/pnas.1101765108 PMC3064331

[27] Plotnik JM, de Waal FBM, Moore D, Reiss D 2010 Self-recognition in the Asian elephant and future directions for cognitive research with elephants in zoological settings. Zoo Biol. 29, 179–191. (doi:10.1002/zoo.20257) 19514017 10.1002/zoo.20257

[28] McGuire M, Raleigh M, Pollack D 1994 Personality features in vervet monkeys: the effects of sex, age, social status, and group composition. Am. J. Primatol. 33, 1–13. (doi:10.1002/ajp.1350330102) 10.1002/ajp.1350330102 31936927

[29] Jenkins SH 2011 Sex differences in repeatability of food-hoarding behaviour of kangaroo rats. Anim. Behav. 81, 1155–1162. (doi:10.1016/j.anbehav.2011.02.021)

[30] King JE, Weiss A, Sisco MM 2008 Aping humans: age and sex effects in chimpanzee (*Pan troglodytes*) and human (*Homo sapiens*) personality. J. Comp. Psychol. 122, 418–427. (doi:10.1037/a0013125) 19014265 10.1037/a0013125

[31] Clubb R, Rowcliffe M, Lee P, Mar KU, Moss C, Mason GJ 2008 Compromised survivorship in zoo elephants. Science 322, 1649 (doi:10.1126/science.1164298) 19074339 10.1126/science.1164298

[32] Yasui S, Konno A, Tanaka M, Idani G, Ludwig A, Lieckfeldt D, Inoue-Murayama M 2013 Personality assessment and its association with genetic factors in captive Asian and African elephants. Zoo Biol. 32, 70–78. (doi:10.1002/zoo.21045) 22996044 10.1002/zoo.21045

[33] IUCN. 2010 IUCN red list of threatened species. See www.iucnredlist.org (accessed 17 May 2016).

[34] Mar KU 2007 The demography and life history strategies of timber elephants in Myanmar. PhD thesis, University College London, London, UK.

[35] Lahdenperä M., Mar KU, Lummaa V 2016 Nearby grandmother enhances calf survival and reproduction in Asian elephants. Sci. Rep. 6, Article number 27213 (doi:10.1038/srep27213) 10.1038/srep27213 PMC4901297 27282468

[36] Freeman HD, Brosnan SF, Hopper LM, Lambeth SP, Schapiro SJ, Gosling SD 2013 Developing a comprehensive and comparative questionnaire for measuring personality in chimpanzees using a simultaneous top-down/bottom-up design. Am. J. Primatol. 75, 1042–1053. (doi:10.1002/ajp.22168) 23733359 10.1002/ajp.22168 PMC3823524

[37] Grand AP, Kuhar CW, Leighty KA, Bettinger TL, Laudenslager ML 2012 Using personality ratings and cortisol to characterize individual differences in African elephants (*Loxodonta africana*). Appl. Anim. Behav. Sci. 142, 69–75. (doi:10.1016/j.applanim.2012.09.002)

[38] Konecná M., Lhota S, Weiss A, Urbánek T, Adamová T, Pluhácek J 2008 Personality in free-ranging Hanuman langur (*Semnopithecus entellus*) males: subjective ratings and recorded behavior. J. Comp. Psychol. 122, 379–389. (doi:10.1037/a0012625) 19014262 10.1037/a0012625

[39] R Core Team. 2015 R: a language and environment for statistical computing. Vienna, Austria: R Foundation for Statistical Computing See https://www.R-project.org/.

[40] Muthén LK, Muthén B 2017 Mplus user's guide, 8th edn.

[41] Gamer M, Lemon J, Fellows I, Singh P 2012 irr: various coefficients of interrater reliability and agreement. See https://cran.r-project.org/web/packages/irr/irr.pdf.

[42] Revelle W 2015 Psych: procedures for personality and psychological research. Evanston, IL: Northwestern University See http://CRAN.R-project.org/package=psych.

[43] Budaev SV 2010 Using principal components and factor analysis in animal behaviour research: caveats and guidelines. Ethology 116, 472–480. (doi:10.1111/j.1439-0310.2010.01758.x)

[44] West SG, Taylor AB, Wu W 2012 Model fit and model selection in structural equation modeling. In Handbook of structural equation modeling (ed. Hoyle RH), pp. 209–231. New York, NY: The Guilford Press.

[45] Brown TA 2015 Confirmatory factor analysis for applied research, 2nd edn New York, NY: The Guilford Press.

[46] McNeish DM 2014 Modeling sparsely clustered data: design-based, model-based, and single-level methods. Psychol. Methods 19, 552–563. (doi:10.1037/met0000024) 25110903 10.1037/met0000024

[47] Raykov T 2004 Behavioral scale reliability and measurement invariance evaluation using latent variable modeling. Behav. Ther. 35, 299–331. (doi:10.1016/S0005-7894(04)80041-8)

[48] Landis JR, Koch GG 1977 The measurement of observer agreement for categorical data. Biometrics 33, 159–174. (doi:10.2307/2529310) 843571

[49] Weiss A, Adams MJ, Widdig A, Gerald MS 2011 Rhesus macaques (*Macaca mulatta*) as living fossils of hominoid personality and subjective well-being. J. Comp. Psychol. 125, 72–83. (doi:10.1037/a0021187) 21341912 10.1037/a0021187 PMC4214372

[50] Muthén B, Muthén LK, Asparouhov T 2016 Regression and mediation analysis using mplus. Los Angeles, CA: Muthén & Muthén.

[51] Dingemanse NJ, De Goede P 2004 The relation between dominance and exploratory behavior is context-dependent in wild great tits. Behav. Ecol. 15, 1023–1030. (doi:10.1093/beheco/arh115)

[52] Rudin FS, Tomkins JL, Simmons LW 2017 Changes in dominance status erode personality and behavioral syndromes. Behav. Ecol. 28, 270–279. (doi:10.1093/beheco/arw151)

[53] Bernstein IS 1981 Dominance relationships and ranks: explanations, correlations and empirical challenges. J. Behav. Brain Sci. 4, 449–453. (doi:10.1017/S0140525X00009857)

[54] Weiss A, Inoue-Murayama M, Hong KW, Inoue E, Udono T, Ochiai T, Matsuzawa T, Hirata S, King JE 2009 Assessing chimpanzee personality and subjective well-being in Japan. Am. J. Primatol. 71, 283–292. (doi:10.1002/ajp.20649) 19199350 10.1002/ajp.20649

[55] McCrae RR, Costa PT. J 2006 Personality in adulthood: a five-factor theory perspective, 2nd edn New York, NY: The Guilford Press.

[56] Clinchy M, Sheriff MJ, Zanette LY 2013 Predator-induced stress and the ecology of fear. Funct. Ecol. 27, 56–65. (doi:10.1111/1365-2435.12007)

[57] Weiss A, Wilson ML, Collins DA, Mjungu D, Kamenya S, Foerster S, Pusey AE 2017 Personality in the chimpanzees of Gombe National Park. Sci. Data 4, 170146 (doi:10.1038/sdata.2017.146) 29064463 10.1038/sdata.2017.146 PMC5654364

[58] Gold KC, Maple TL 1994 Personality assessment in the gorilla and its utility as a management tool. Zoo Biol. 13, 509–522. (doi:10.1002/zoo.1430130513)

[59] Uher J 2008 Three methodological core issues of comparative personality research. Eur. J. Pers. 22, 475–496. (doi:10.1002/per.688)

[60] Gosling SD, John OP 1999 Personality dimensions in nonhuman animals: a cross-species review. Curr. Dir. Psychol. Sci. 8, 69–75. (doi:10.1111/1467-8721.00017)

[61] Mullan SM, Main DC 2007 Behaviour and personality of pet rabbits and their interactions with their owners. Vet. Rec. 160, 516–520. (doi:10.1136/vr.160.15.516) 17435098 10.1136/vr.160.15.516

[62] Kis A, Turcsán B, Miklósi Á, Gácsi M 2012 The effect of the owner's personality on the behaviour of owner-dog dyads. Interact. Stud. 13, 373–385. (doi:10.1075/is.13.3.03kis)

[63] English S, Nakagawa S, Clutton-Brock TH 2010 Consistent individual differences in cooperative behaviour in meerkats (*Suricata suricatta*). J. Evol. Biol. 23, 1597–1604. (doi:10.1111/j.1420-9101.2010.02025.x) 20492087 10.1111/j.1420-9101.2010.02025.x

[64] Coleman K, Tully LA, Mcmillan JL 2005 Temperament correlates with training success in adult rhesus macaques. Am. J. Primatol. 65, 63–71. (doi:10.1002/ajp.20097) 15645460 10.1002/ajp.20097

